# Bioinformatics and pathway enrichment analysis identified hub genes and potential biomarker for gastric cancer prognosis

**DOI:** 10.3389/fonc.2023.1187521

**Published:** 2023-06-09

**Authors:** Elham Darang, Zahra Pezeshkian, Seyed Ziaeddin Mirhoseini, Shahrokh Ghovvati

**Affiliations:** ^1^ Department of Animal Sciences, Faculty of Agriculture, University of Guilan, Rasht, Guilan, Iran; ^2^ Research and Development Center (R&D), BioGenTAC Inc., Rasht, Guilan, Iran

**Keywords:** bioinformatic analysis, biomarker, gastric cancer, hub genes, meta-analysis

## Abstract

**Introduction:**

Gastric cancer is one of the most common cancers in the world. This study aimed to identify genes, biomarkers, and metabolic pathways affecting gastric cancer using bioinformatic analysis and meta-analysis.

**Methods:**

Datasets containing gene expression profiles of tumor lesions and adjacent non-tumor mucosa samples were downloaded. Common differentially expressed genes between data sets were selected to identify hub genes and further analysis. Gene Expression Profiling and Interactive Analyses (GEPIA) and the Kaplan-Meier method were used to further validate the expression level of genes and plot the overall survivalcurve, respectively.

**Results and disscussion:**

KEGG pathway analysis showed that the most important pathway was enriched in ECM-receptor interaction. Hub genes includingCOL1A2, FN1, BGN, THBS2, COL5A2, COL6A3, SPARC and COL12A1 wereidentified. The top interactive miRNAs including miR-29a-3p, miR-101-3p,miR-183-5p, and miR-15a-5p targeted the most hub genes. The survival chart showed an increase in mortality in patients with gastric cancer, which shows the importance of the role of these genes in the development of the disease and can be considered candidate genes in the prevention and early diagnosis of gastric cancer.

## 1 Introduction

Gastric cancer (GC) is the fifth most common cancer in the world and the fourth leading cause of cancer death (1). In addition to non-genetic factors such as smoking, alcohol consumption, poor diet, physical inactivity, viral infections, and stress, genetic factors are also other contributing factors to this cancer (2).

To date, the only treatment for GC is surgery and chemotherapy (3). Because early-stage GC is usually asymptomatic and diagnosis is often made in the advanced stage of the disease. Using the bioinformatics technique is an effective way to identify genes and their protein products as biomarkers. In this way, markers can be examined in the blood of patients in the early stages, and then diagnostic tests can be performed (1, 4).

Microarray allows the simultaneous examination of tens to thousands of RNAs in an organism or a cell (5). Analysis of the expression level of this number of genes reveals the processes that take place simultaneously in the cell. Also, comparing the expression level of genes in both healthy and sick conditions provide valuable studies of how the disease originated and progressed (6).

Despite being a high-throughput method for gene expression analysis, microarray has some limitations that are generally related to reproducibility and sensitivity to technical and computational errors (7). Integrating already existing information can increase the reproducibility and reliability of results. The statistical technique of integrating different but related studies is called meta-analysis, which makes it possible to identify common genes and understand common molecular mechanisms. Integrated analysis of biological information helps identify and screen cancer-related genes and develop new treatment strategies for disease management (8). In this study, two gene expression profiles in the GEO database containing samples from GC tumor tissue and adjacent tissue were used to identify common genes and biomarkers and metabolic pathways affecting GC.

## 2 Materials and methods

### 2.1 Data collection

Gene expression data were downloaded from the public database of the Gene Expression Omnibus (GEO) database of NCBI (http://www.ncbi.nlm.nih.gov/geo/). The first dataset (GEO accession number GSE79973) contained gene expression profiling of 10 samples of tumor lesions and 10 samples of adjacent non-tumor mucosa. The second dataset (GEO accession number GSE19826) contained gene expression profiling of 12 samples of tumor lesions and 12 samples of adjacent non-tumor mucosa numbers. The gene expression profiling was generated by the Affymetrix platform.

### 2.2 Identification of differentially expressed genes in GC and database integration

We applied the GEO2R analysis to identify differentially expressed genes (DEGs) with Benjamini–Hochberg correction to control the false discovery rate. Cut-off criteria for sorting significant DEGs were p-value < 0.05 and |log2 fold change (FC)| ≥ 1. Finally, the mentioned analysis was applied to both selected datasets, and differentially expressed genes with different expression were obtained for both datasets. To integrate the data, common differentially expressed genes in both datasets were selected for further analyses.

### 2.3 Gene ontology and pathway enrichment analysis

Database for Annotation, Visualization, and Integrated Discovery (DAVID) was used to interpret the list of DEGs. Pathway analysis and gene ontology (GO) analysis were performed using DAVID. Biological pathways with a p-value less than 0.05 were considered significant. The outcomes of DAVID were then imported into the GO plot in R Studio. The GO Bubble plot was used to visualize the functional enrichment of DEGs, which facilitates the combination of expression data with functional assessment outcomes. DEGs were exposed to Clue GO v2.5.7 to perform and visualize GO analysis. The p-values <0.05 were considered significant.

### 2.4 PPI network and cytohubba analysis

The Search Tool for the Retrieval of Interacting Genes/Proteins (STRING) database was used to study the protein products of the genes. This database provides networks containing regulatory connections between genes as output. Cytohubba is a simple Cytoscape plugin that ranks the importance of nodes in the PPI network with different algorithms for identifying key biological elements. In this study, using the degree algorithm, 8 genes with a rank higher than 7 were selected as hub genes.

### 2.5 Survival analysis and confirmation of hub gene

Using the Kaplan-Meier plotter (http://kmplot.com/analysis/), Kaplan-Meier diagrams of the top 8 hub genes were drawn. The Kaplan-Mahir estimator is an estimator for estimating the survival function from survival information.

Hub genes and their protein products were evaluated by Gene Expression Profiling Interactive Analysis (GEPIA) web tool. GEPIA is a web-based tool for providing fast and customizable functions based on TCGA and GTEx data (http://gepia.cancer-pku.cn). This database was also used to plot survival and gene expression diagrams in a box plot.

### 2.6 miRNAs-mRNA network construction

The Encyclopedia RNA Interactomes (ENCORI, http://starbase.sysu.edu.cn/) is a public platform that detects more than 2.5 million miRNA-mRNA interactions. The target miRNAs of the hub genes were screened based on criteria of SLIP-DATA ≥ 5, Degradome-Data ≥ 0, pan-Cancer ≥ 6, and programNum ≥ 3 using the Encyclopedia RNA Interactomes. In addition, the miRNA-hub gene network was created by Cytoscape software.

### 2.7 Drug-hub gene interaction

Interactions between hub genes and related therapeutic drugs were examined through the drug-gene interaction database (http://www.dgidb.org/search interactions). The drug-gene interaction database is a web-based source of information on gene-drug interactions. We also mapped the interactive network between hub genes and effective drugs using the STITCH online tool (http://stitch.embl.de/cgi).

## 3 Results

### 3.1 Identification of common differentially expressed genes in two datasets

**Figure 1:**
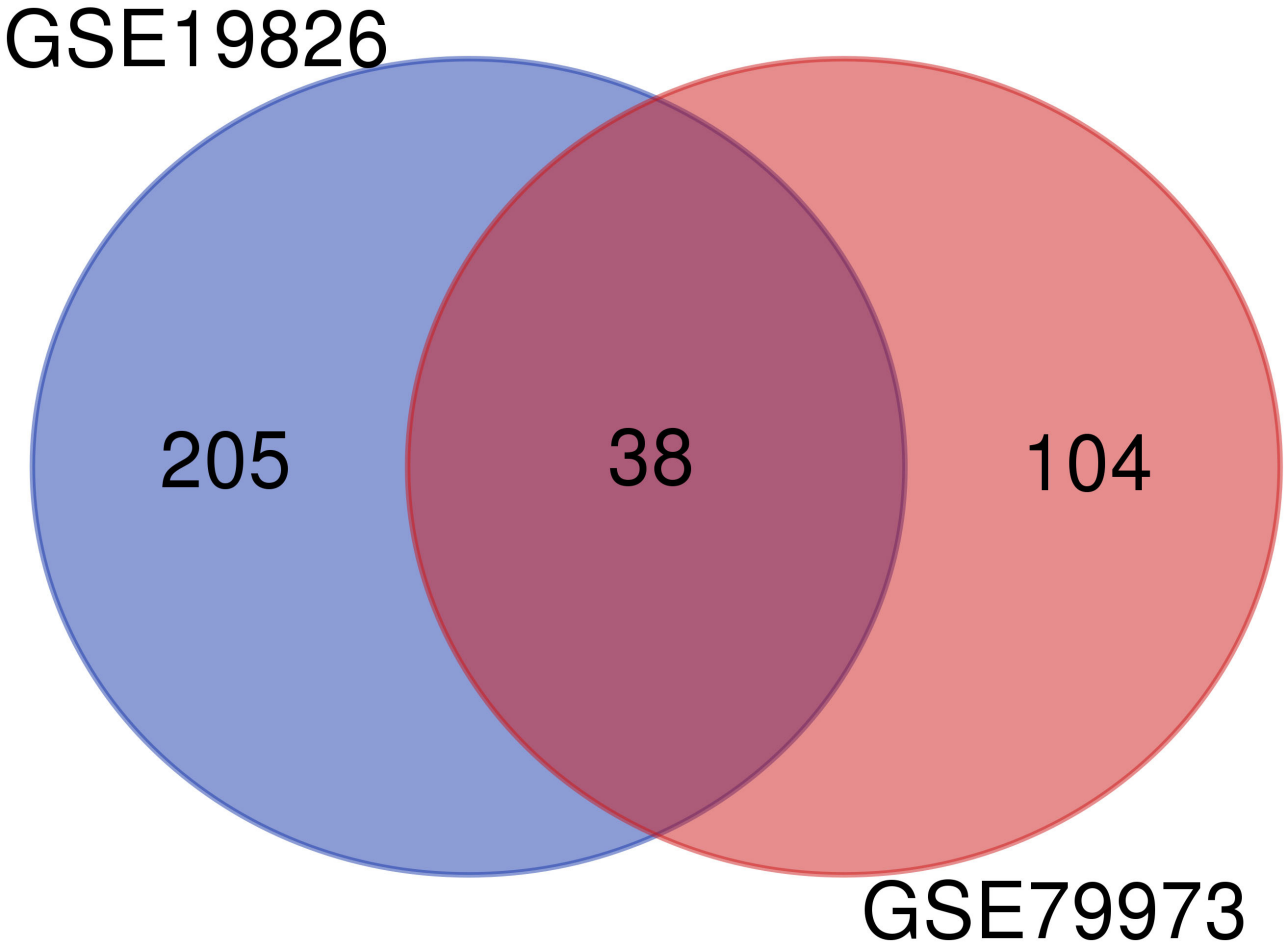
Common genes between GSE79973 and GSE19826.

To obtain the DEGs, we analyzed the GSE79973 and GSE19826 datasets separately using GEO2R. We investigated common DEGs between two datasets. Finally, 38 common genes were found between these two datasets (**Figure 1**).

### 3.2 DAVID enrichment analysis for common differentially expressed genes

To understand the functions and biological significance of the mutual DEGs, we performed an enrichment analysis of DEGs in terms of biological process (BP), molecular function (MF), and cellular component (CC) (**Table 1**). Sixteen significantly enriched BP terms were found, the most significant of which were “collagen catabolic process”, “extracellular matrix organization”, and “skeletal system development”. Ten significant CC terms were identified, the most significant of which were “proteinaceous extracellular matrix”, “collagen trimer”, and “extracellular matrix”. The significantly enriched MF terms were “extracellular matrix structural constituent”, “integrin binding”, “calcium ion binding”, and “heparin binding”. KEGG pathway enrichment analysis revealed the association of the DEGs in 5 pathways including “Protein digestion and absorption”, “ECM-receptor interaction”, “Focal adhesion”, “Amoebiasis”, and “PI3K-Akt signaling pathway” (**Table 2**).

**Table 1:** The significant Gene Ontology terms enriched by the mutually differentially expressed genes in GSE79973 and GSE19826.

Category	GO ID	GO Terms	P-Value	Genes
Biological process	GO:0030574	collagen catabolic process	4.13E-11	COL18A1, ADAMTS2, COL1A2, COL12A1, COL5A2, COL10A1, COL6A3, COL8A1
	GO:0030198	extracellular matrix organization	1.36E-10	COL18A1, VCAN, SPARC, COL1A2, COL5A2, BGN, FN1, COL10A1, COL6A3, COL8A1
	GO:0001501	skeletal system development	9.23E-09	HOXA10, TEAD4, VCAN, COL1A2, CDH11, COL12A1, COL5A2, COL10A1
	GO:0007155	cell adhesion	1.54E-08	COL18A1, AZGP1, VCAN, CDH11, COL12A1, FN1, COL6A3, COL8A1, THY1, THBS2, WISP1
	GO:0035987	endodermal cell differentiation	2.15E-05	COL12A1, FN1, COL8A1, INHBA
	GO:0030199	collagen fibril organization	6.59E-05	ADAMTS2, COL1A2, COL12A1, COL5A2
	GO:0001525	angiogenesis	1.03E-03	COL18A1, SRPX2, FN1, COL8A1, THY1
	GO:0002576	platelet degranulation	1.17E-03	SPARC, ACTN1, FN1, TIMP1
	GO:0051216	cartilage development	6.33E-03	COL10A1, TIMP1, SULF1
	GO:0016525	negative regulation of angiogenesis	6.97E-03	SPARC, THBS2, SULF1
	GO:0001503	ossification	1.14E-02	SPARC, CDH11, COL5A2
	GO:0042060	wound healing	1.14E-02	SPARC, FN1, TIMP1
	GO:0030208	dermatan sulfate biosynthetic process	2.40E-02	VCAN, BGN
	GO:0030207	chondroitin sulfate catabolic process	2.80E-02	VCAN, BGN
	GO:0048041	focal adhesion assembly	4.75E-02	ACTN1, THY1
	GO:0030206	chondroitin sulfate biosynthetic process	4.94E-02	VCAN, BGN
Cellular component	GO:0005578	proteinaceous extracellular matrix	1.07E-13	COL18A1, SPARC, BGN, FN1, WISP1, ADAMTS2, VCAN, COL1A2, COL5A2, COL6A3, COL10A1, TIMP1, CTHRC1
	GO:0005581	collagen trimer	6.03E-10	COL18A1, COL1A2, COL12A1, COL5A2, COL10A1, COL6A3, TIMP1, CTHRC1
	GO:0031012	extracellular matrix	5.82E-09	COL18A1, VCAN, COL1A2, COL12A1, COL5A2, BGN, FN1, COL6A3, COL8A1, THBS2
	GO:0005615	extracellular space	1.97E-08	COL18A1, PSAPL1, SPARC, ACTN1, COL12A1, FN1, SULF1, WISP1, AZGP1, VCAN, PPFIBP2, SRPX2, COL1A2, COL6A3, TIMP1, CTHRC1
	GO:0005576	extracellular region	2.74E-08	COL18A1, SPARC, ACTN1, COL12A1, BGN, FN1, INHBA, THBS2, ADAMTS2, AZGP1, VCAN, COL1A2, COL5A2, COL6A3, COL8A1, COL10A1, TIMP1
	GO:0005788	endoplasmic reticulum lumen	2.24E-06	COL18A1, COL1A2, COL12A1, COL5A2, COL10A1, COL6A3, COL8A1
	GO:0031093	platelet alpha granule lumen	1.88E-04	SPARC, ACTN1, FN1, TIMP1
	GO:0070062	extracellular exosome	2.03E-04	CLIC6, COL18A1, ACTN1, COL12A1, BGN, FN1, THY1, SVIP, PBLD, AZGP1, COL1A2, CDH11, COL6A3, COL8A1, TIMP1, MEST
	GO:0005604	basement membrane	5.48E-04	COL18A1, SPARC, TIMP1, THBS2
	GO:0031091	platelet alpha granule	2.81E-02	SPARC, THBS2
Molecular function	GO:0005201	extracellular matrix structural constituent	3.57E-04	VCAN, COL1A2, COL5A2, BGN
	GO:0005178	integrin binding	1.32E-03	ACTN1, FN1, THY1, WISP1
	GO:0042802	identical protein binding	4.03E-03	TRIM50, COL18A1, SRPX2, COL1A2, FN1, INHBA, PBLD
	GO:0005509	calcium ion binding	1.54E-02	VCAN, SPARC, ACTN1, CDH11, THBS2, SULF1
	GO:0005539	glycosaminoglycan binding	3.67E-02	VCAN, BGN
	GO:0008201	heparin binding	4.33E-02	FN1, THBS2, WISP1

**Table 2:** KEGG pathway analysis of differentially expressed genes (p<0.05).

Category	GO ID	GO Term	P-Value	Genes
KEGG_PATHWAY	hsa04974	Protein digestion and absorption	2.30E-06	COL18A1, COL1A2, COL12A1, COL5A2, COL10A1, COL6A3
KEGG_PATHWAY	hsa04512	ECM-receptor interaction	6.38E-05	COL1A2, COL5A2, FN1, COL6A3, THBS2
KEGG_PATHWAY	hsa04510	Focal adhesion	1.43E-04	COL1A2, ACTN1, COL5A2, FN1, COL6A3, THBS2
KEGG_PATHWAY	hsa05146	Amoebiasis	2.45E-03	COL1A2, ACTN1, COL5A2, FN1
KEGG_PATHWAY	hsa04151	PI3K-Akt signaling pathway	1.09E-02	COL1A2, COL5A2, FN1, COL6A3, THBS2

### 3.3 Identification of hub proteins

**Figure 2:**
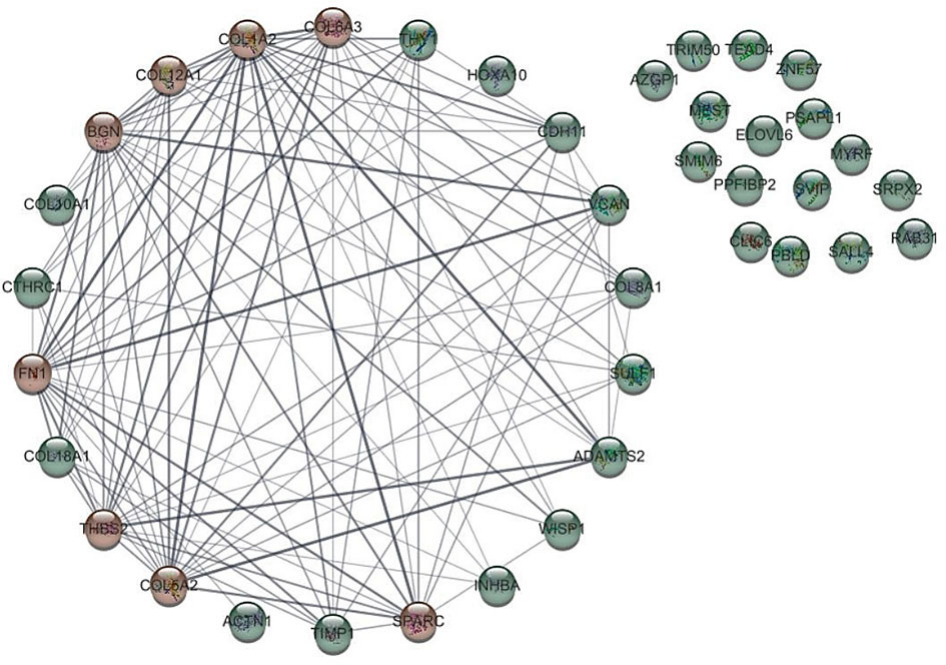
Protein-protein interaction of genes with different expression (DEGs) with other genes. The hub gene is marked in red.

Biomolecules in biological systems work by interacting with each other. Therefore, to gain insight into the functional interaction of DEGs, a substantial protein-protein interaction (PPI) network was formed using the STRING dataset for the common proteins of datasets (**Figure 2**). PPI network using cyto-Hubba plugin in Cytoscape software showed that 8 genes including *COL1A2, FN1, BGN, THBS2, COL5A2, COL6A3, SPARC* and *COL12A1*, which were known as hub genes, had the greatest effect compared to other genes.

### 3.4 Survival analysis and validation of hub genes

**Figure 3:**
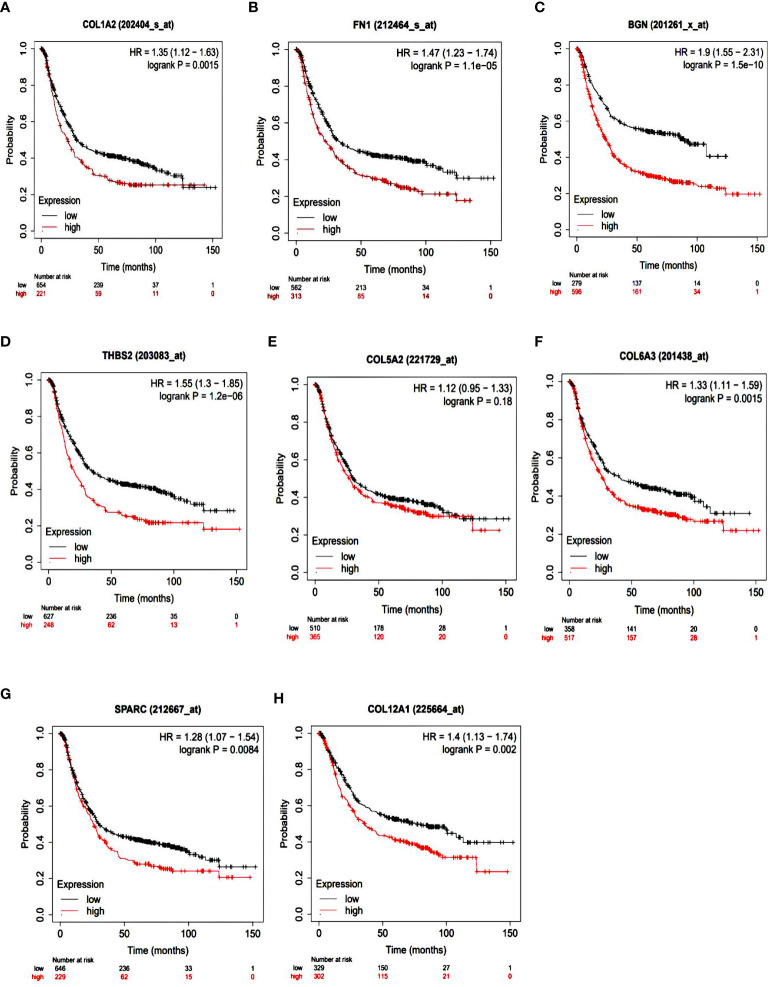
Kaplan-Meier overall survival analyses of patients with gastric cancer based on expression of the eight key genes. **(A)** COL1A2; **(B)** FN1; **(C)** BGN; **(D)** THBS2; **(E)** COL5A2; **(F)** COL6A3; **(G)** SPARC; **(H)** COL12A1.

**Figure 4:**
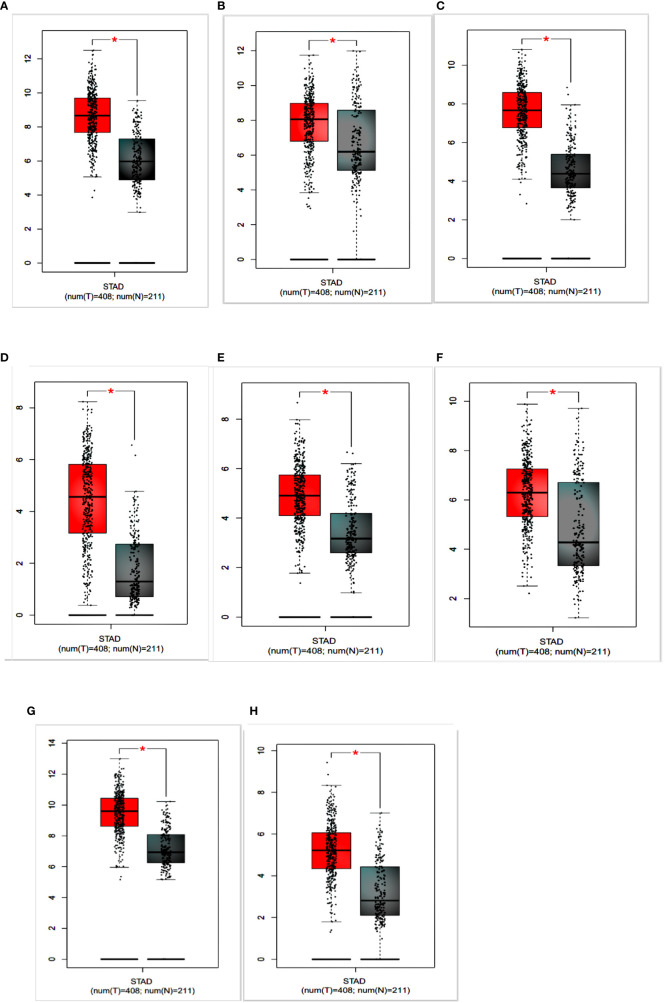
Expression of hub genes in gastric cancer patients and healthy individuals is shown in a box plot. **(A)** COL1A2; **(B)** FN1; **(C)** BGN; **(D)** THBS2; **(E)** COL5A2; **(F)** COL6A3; **(G)** SPARC; **(H)** COL12A1.

To identify the prognostic value of the eight hub genes, overall survival curves based on expression were plotted using the Kaplan-Meier method (**Figure 3**). The curves showed that overexpression of seven key genes (*COL1A2, FN1, BGN, THBS2*, *COL6A3, SPARC, COL12A1*) was associated with reduced overall survival time in GC patients. This difference was not significant for the *COL5A2* gene. For more accurate validation of the hub genes, and in particular the links between protein networks, the final genes were examined in the GEPIA web tool. Gene expression diagrams were plotted as box diagrams (**Figure 4**). The expression of all hub genes was higher in GC patients than in healthy subjects, but among them, *BGN* and *THBS2* showed the highest increase in expression, while the difference in expression was the lowest in *COL6A3* and *FN1*.

### 3.5 miRNA–mRNA network

**Figure 5:**
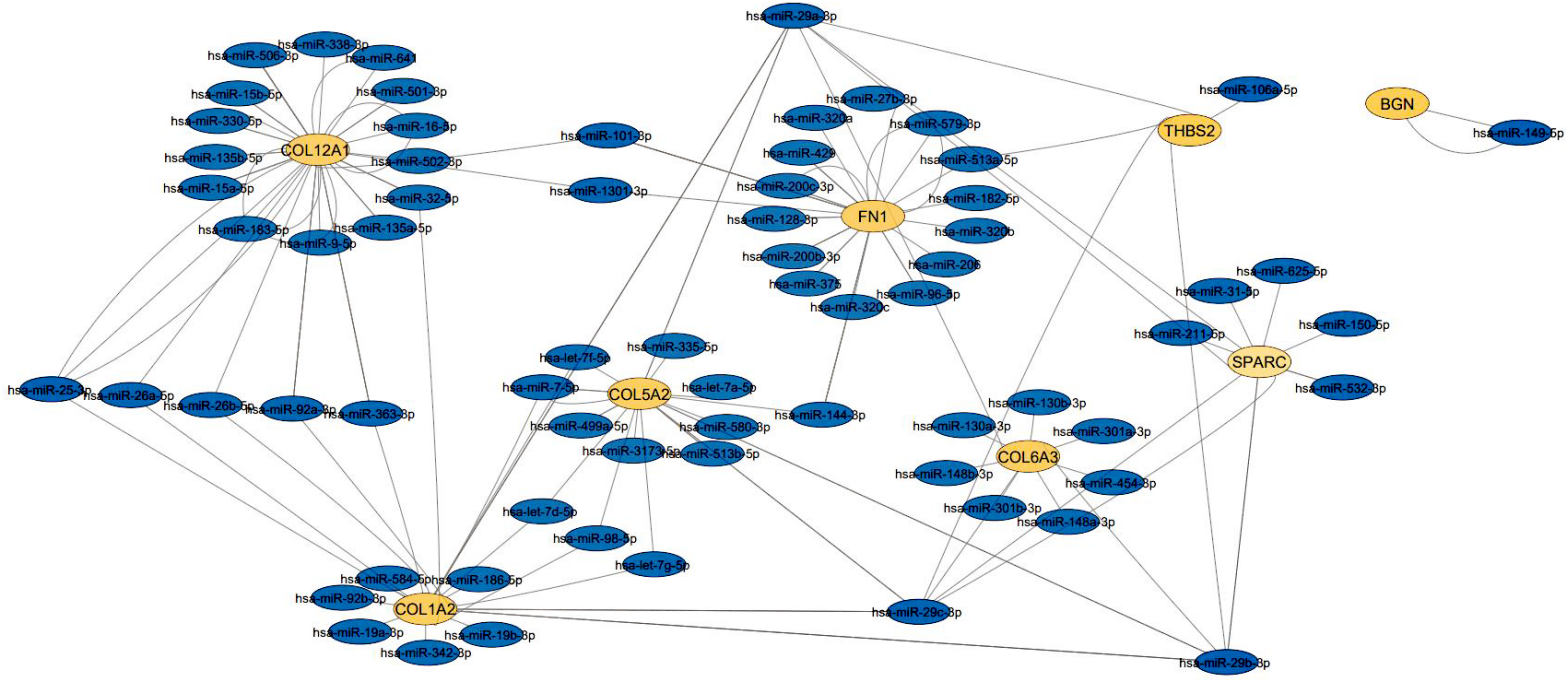
The interaction network between hub genes and target miRNAs. Hub genes are presented in orang circles, whereas target miRNAs are shown in blue circles.

The exact relationship between hub genes and miRNA was determined (**Table 3**) and the miRNA-hub gene interaction network consisting of 8 hub genes and 98 miRNA was formed by cytoscape software (**Figure 5**). The four interactive hub genes that most miRNAs target were: *COL12A1* (grade = 47), *FN1* (grade = 34), *COL1A2* (grade = 26), and *COL5A2* (grade = 22). In addition, hsa-hsa-miR-29a-3p (grade = 9), hsa-miR-101-3p (grade = 5), hsa-miR-183-5p (grade = 4), hsa-miR-15a-5p (degree, score = 3) were the top four interactive miRNAs that targeted the most hub genes.

**Table 3:** The respective miRNAs targeting the 8 hub genes.

Gen	miRNA name
BGN	hsa-miR-149-5p,
COL12A1	hsa-miR-101-3p,hsa-miR-1301-3p,hsa-miR-135a-5p,hsa-miR-135b-5p,hsa-miR-15a-5p,hsa-miR-15b-5p,hsa-miR-16-5p,hsa-miR-183-5p,hsa-miR-25-3p,hsa-miR-26a-5p,hsa-miR-26b-5p,hsa-miR-32-5p,hsa-miR-330-5p,hsa-miR-338-3p,hsa-miR-363-3p,hsa-miR-501-3p,hsa-miR-502-3p,hsa-miR-506-3p,hsa-miR-641,hsa-miR-92a-3p,hsa-miR-9-5p,
COL1A2	hsa-let-7d-5p,hsa-let-7f-5p,hsa-let-7g-5p,hsa-miR-186-5p,hsa-miR-19a-3p,hsa-miR-19b-3p,hsa-miR-25-3p,hsa-miR-26a-5p,hsa-miR-26b-5p,hsa-miR-29a-3p,hsa-miR-29b-3p,hsa-miR-29c-3p,hsa-miR-32-5p,hsa-miR-342-3p,hsa-miR-363-3p,hsa-miR-584-5p,hsa-miR-7-5p,hsa-miR-92a-3p,hsa-miR-92b-3p,hsa-miR-98-5p,
COL5A2	hsa-let-7a-5p,hsa-let-7d-5p,hsa-let-7f-5p,hsa-let-7g-5p,hsa-miR-144-3p,hsa-miR-29b-3p,hsa-miR-29c-3p,hsa-miR-3173-5p,hsa-miR-335-5p,hsa-miR-499a-5p,hsa-miR-513b-5p,hsa-miR-580-3p,hsa-miR-7-5p,hsa-miR-98-5p,
COL6A3	hsa-miR-130a-3p,hsa-miR-130b-3p,hsa-miR-148a-3p,hsa-miR-148b-3p,hsa-miR-29a-3p,hsa-miR-29b-3p,hsa-miR-29c-3p,hsa-miR-301a-3p,hsa-miR-301b-3p,hsa-miR-454-3p,
FN1	hsa-miR-101-3p,hsa-miR-128-3p,hsa-miR-1301-3p,hsa-miR-144-3p,hsa-miR-182-5p,hsa-miR-200b-3p,hsa-miR-200c-3p,hsa-miR-206,hsa-miR-27b-3p,hsa-miR-320a,hsa-miR-320b,hsa-miR-320c,hsa-miR-375,hsa-miR-429,hsa-miR-513a-5p,hsa-miR-579-3p,hsa-miR-96-5p,
SPARC	hsa-miR-150-5p,hsa-miR-211-5p,hsa-miR-29a-3p,hsa-miR-29b-3p,hsa-miR-29c-3p,hsa-miR-31-5p,hsa-miR-532-3p,hsa-miR-625-5p,
THBS2	hsa-miR-106a-5p,hsa-miR-29a-3p,hsa-miR-29b-3p,hsa-miR-29c-3p,hsa-miR-513a-5p,

### 3.6 Drug-gene interaction analysis

**Figure 6:**
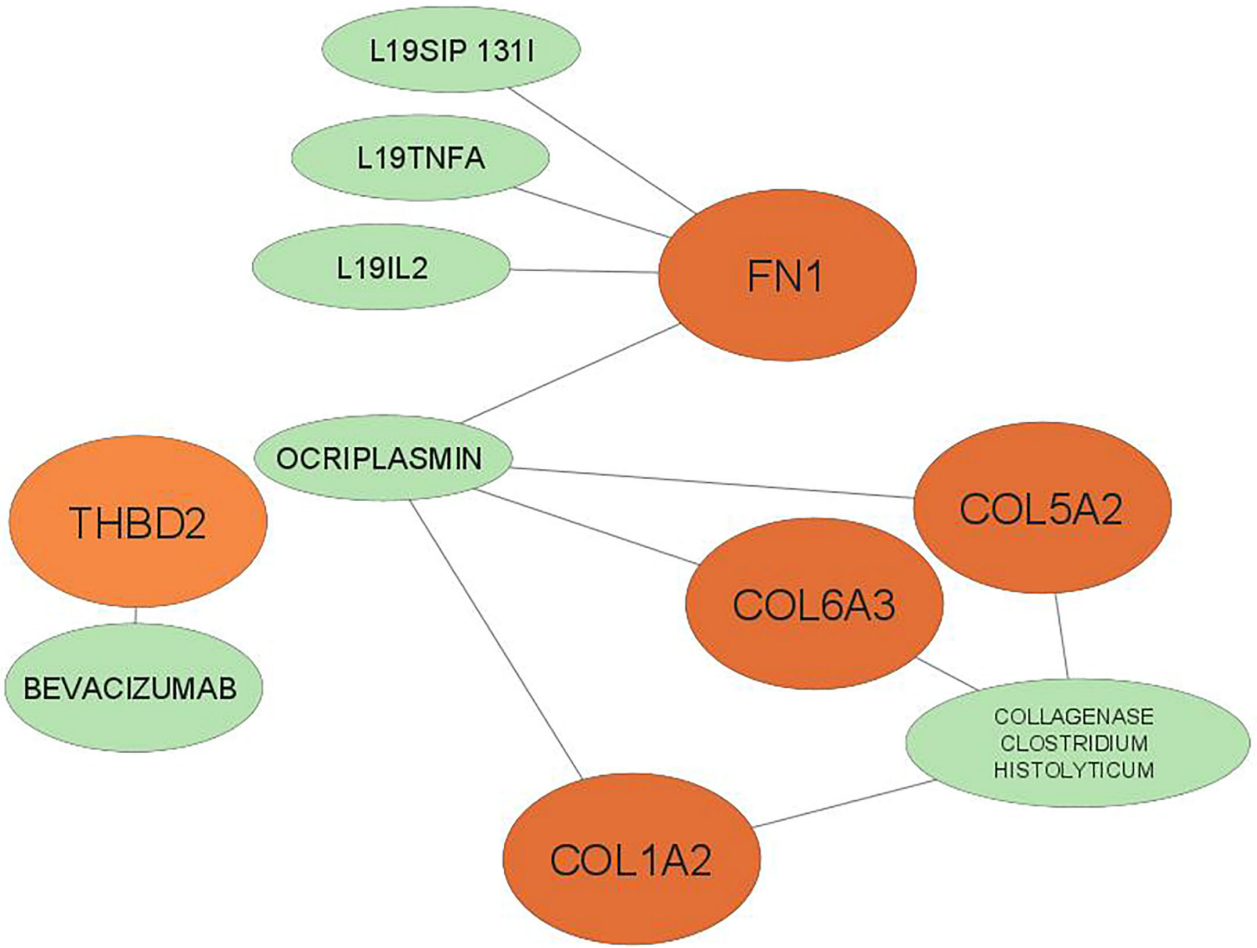
The 6 predicted drugs associated with the hub genes. orange represents hub genes and green represents potential drugs. BGN, SPARC No interactions found.

Drugs were ranked based on the highest total score in the drug-gene interaction database (**Table 4**). A total of 6 drugs related to 5 key genes *(FN1, COL1A2, THBD2, COL6A3, COL15A2)* were found and the network of drugs and hub genes was plotted using Cytoscape (**Figure 6**). No associated drugs were found for *BGN* and *SPARC* genes.

**Table 4:** Top 7 drugs by score ranking in DGIdb.

GENE	Drug	Interaction Type & Directionality	Interaction Score
FN1	L19IL2	n/a	15.95
FN1	L19TNFA	n/a	15.95
FN1	L19SIP 131I	n/a	15.95
THBD2	BEVACIZUMAB	n/a	3.87
COL1A2	COLLAGENASE CLOSTRIDIUM HISTOLYTICUM	n/a	2.28
FN1	OCRIPLASMIN	cleavage (inhibitory)	1.29
COL5A2	COLLAGENASE CLOSTRIDIUM HISTOLYTICUM	n/a	1.14
COL6A3	COLLAGENASE CLOSTRIDIUM HISTOLYTICUM	n/a	1.14
COL1A2	OCRIPLASMIN	n/a	0.86
COL5A2	OCRIPLASMIN	n/a	0.86
COL6A3	OCRIPLASMIN	n/a	0.86

## 4 Discussion

Today, early detection of cancer and its effective treatment are critical in the recovery, diagnosis and management of cancer. Therefore, it is necessary to identify sensitive and specific methods of diagnosis in the early stages. As a result, the knowledge of cancer at the molecular level has greatly increased, and this has led to the use of targeted therapies for cancer. The discovery of biomarkers from microarray data is an important goal in molecular medicine and has wide clinical applications. Biomarkers have a wide range of applications in the early diagnosis of the disease, the diagnosis of the disease stage, the study of the treatment process and the prediction of cancer recurrence.

In the present study, two datasets with the Affymetrix Human Genome joint platform were used. Each data set included a comparison of gastric tumor tissue samples with adjacent normal tissue. Using commonalities over a set of data, which is a meta-analysis approach to data analysis, provides highly reliable results for future analysis. Meta-analysis showed that 38 genes in both datasets were differently expressed between the two GC and adjacent normal tissues, and further analyzes were performed on these common differentially expressed genes. The results of GO analysis in biological process enrichment showed that these common DEGs were more significantly enriched in skeletal system development, negative regulation of angiogenesis, collagen fibril organization, and cartilage development. This was similar to the results reported by Wu et al. (2019) who reported that DEGs were riched in the biological processes of collagen fibril organization and skeletal system development through the integration of five microarray datasets related to GC (9).

KEGG pathway analysis of DEGs showed that the most important pathway was enriched in ECM-receptor interaction and most genes (6 genes) were involved in protein digestion and absorption and focal adhesion. The extracellular matrix (ECM) consists of proteoglycans (collagen and elastin), fibrous proteins (lumican and decorin) and binding proteins (fibronectin and vitronectin) and also acts as a reservoir of CTGF, β-TGF and other growth factors (10). As a key component, ECM plays a role in processes such as cell division, cell migration, differentiation, carcinogenesis, and apoptosis (11–13).

Cao et al. (2018) showed that most DEGs were enriched in three mRNA expression profiles associated with GC in ECM, collagen catabolic process, fibrillar collagen organization, and cell adhesion. They also reported that three KEGG pathways, including receptor interaction - ECM, protein digestion and absorption, and focal adhesion pathways were significantly enriched (14). Li et al. (2020) who analyzed four GEO datasets from the GC population of China, found similar results (13).

Using identified DEGs between gastric cancerous tissue and healthy gastric tissue, Jiang et al. (2014) reported that the ECM receptor interaction pathway identified in multiple cancers also plays a key role in GC biology (15).

Gene network analysis of 38 differentially expressed genes with 38 nodes and 107 edges showed that 16 genes had no relationship with each other and other genes in the network, while 8 genes including *COL1A2, FN1, BGN, COL5A2, THBS2*, *COL6A3, SPARC, COL12A1*, and *COL1A2*, showed the highest degree of connection, respectively. Since the higher the degree of binding of a gene in the network, the greater its association with GC (16), these 8 genes were identified as hub genes.

Hub gene survival analysis was performed and showed that increasing the expression of seven hub genes *COL1A2, FN1, BGN, THBS2, COL6A3, SPARC*, and *COL12A1* significantly reduced the survival of GC patients, but this relationship was not significant for *COL5A2* gene. This was in contrast to the results of Liu et al. (2018) who reported that down expression of the *COL5A2* gene indicated better overall survival in patients with the disease (17). The results of the study of the expression of hub genes in tumors and normal tissues using GEPIA confirms the increased expression of these genes in GC.

**Figure 7:**
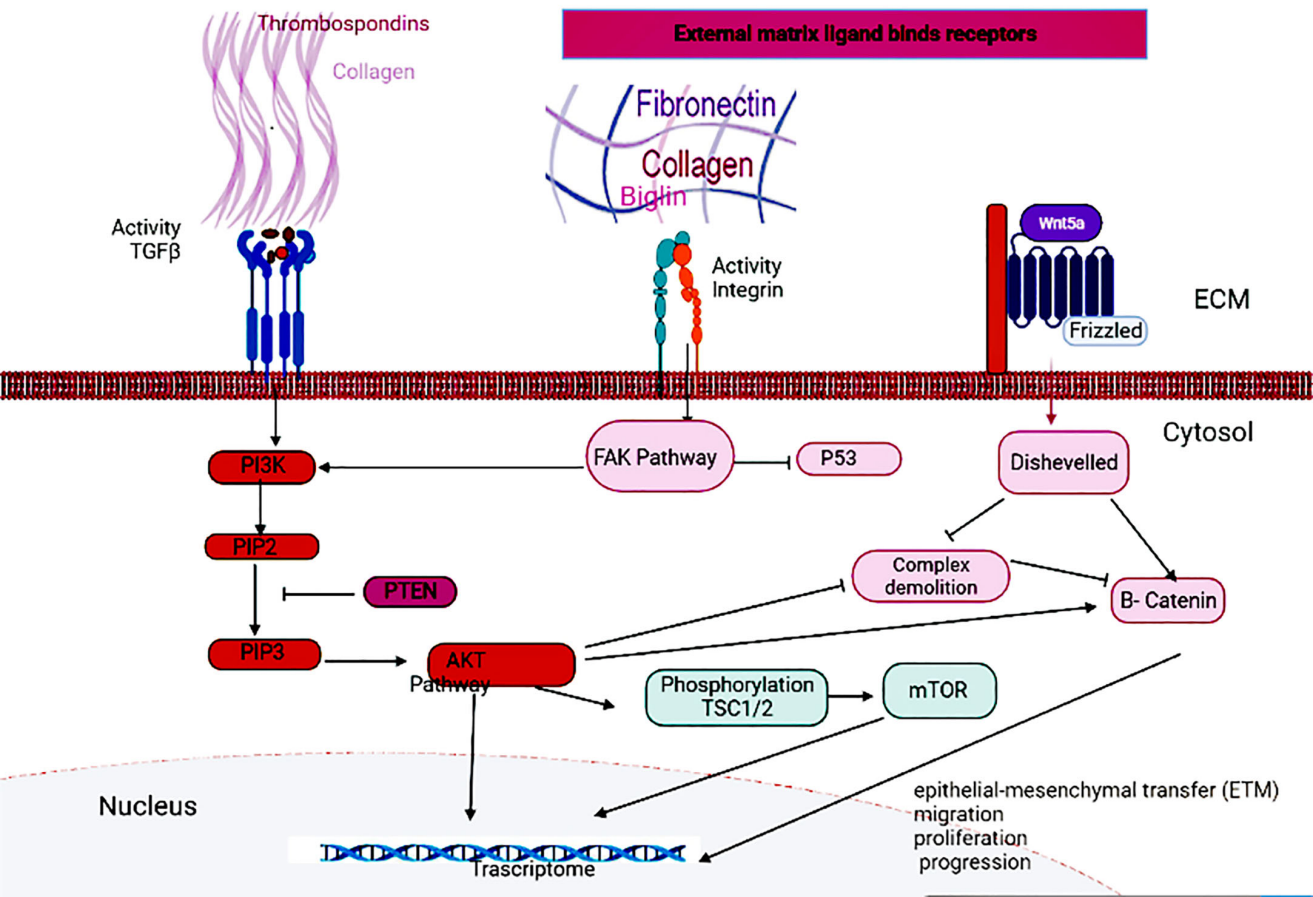
Signaling pathways related to GC.

Different signaling pathways of intracellular messengers are involved in the proliferation, invasion and metastasis of cancers. The binding of growth factors and cytokines to the TGFβ receptor activates PI3K and activates the Akt pathway. Akt can activate β-catenin by stimulating nuclear transfer or degradation complex degradation, thereby, directly and indirectly, affecting the Wnt pathway (18, 19). Akt also inhibits TSC1/2 through phosphorylation and activates the mTOR pathway (19, 20). Phosphatase and tensin homolog (PTEN) can inhibit the activation of the AKT pathway by acting on Phosphatidylinositol-3,4,5-triphosphate (PIP3) and converting it to Phosphatidylinositol 4,5-bisphosphate (PIP2) (21). Integrins are also other receptors that can send extracellular signals to Focal adhesion kinase (FAK). Activation of the Wnt pathway also leads to the activation of the disheveled protein, resulting in the degradation of the degradation complex, as well as allowing the production of dephosphorylated beta-catenin and its migration to the nucleus (22). Hub genes can affect the proliferation and invasion of GC cells and transcriptional activation by acting on either of these pathways (**Figure 7**).

The *COL1A2, COL5A2, COL6A3*, and *COL12A1* genes provide instructions for making a component of type I, V, VI, and XII collagen, respectively, and participate in the formation of collagen in extracellular matrix proteins (13). Some collagen proteins are associated with the progression and prognosis of cancer. These collagens stimulate the transfer of tumor cells (23, 24).

A study reported that *COL1A2* gene mRNA expression in malignant gastric tissues was significantly higher than in normal malignant tissues (25). Increased *COL1A2* expression has also been reported in many types of cancer, including breast cancer, cervical cancer, and colon cancer (26). The expression of *COL1A2, COL6A3*, and *THBS2* genes leads to the development, migration, and invasion of gastric cancer cells, and reduces apoptosis through the PI3K-Akt pathway (16, 27). mir 129-5p can reduce the expression of *COL1A1* and thus inhibit the proliferation, invasion, and migration of GC cells (28).

A bioinformatic analysis study has shown that *COL5A2* is a candidate biomarker of GC (29). Also Alpha 2 collagen chain V plays an important role in the development of colorectal cancer, breast tumors, and osteosarcoma (30). Research shows that increased expression of *COL5A2* is associated with increased expression of cytokines such as VEGF, which lead to the growth of tumor cells and unrestricted angiogenesis (30).

Through microarray meta-analysis, Xie et al. (2014) showed that *COL6A3* is overexpressed in GC (31). Collagen VI can directly affect tumor cells and increase tumorigenesis by activating the Akt – GSK-3b – β-catenin – TCF/LEF pathway and positively regulating transcription factors (TFs), protein kinases, angiogenic factors, and growth factors (32). Collagen VI also promotes resistance to chemotherapy through 1F/1E (MT1F/1E). The conversion of tumor cells from an epithelial phenotype to a mesenchymal phenotype is the epithelial-mesenchymal transfer (EMT), which is a sign of tumor migration and proliferation. Endotrophin (ETP), a peptide isolated from collagen VI, targets tumor cells via the TGF-b-dependent pathway to induce EMT and fibrosis. It causes tumor inflammation through macrophages and increases the expression of TNF-a and IL-6, and also increases angiogenesis with high expression of *CD31*, *VEGFR2*, and *HIF1a* (33).

Wu et al. (2020) reported that there is a negative association between *COL12A1* methylation and colorectal cancer (34). *COL12A1* alters the structural components of the extracellular matrix by using some of the kinases, miRNAs, and transcription factors associated with cancer and by integrin binding and collagen binding (34, 35).

Fibronectin 1 (*FN1*) is part of the extracellular matrix (ECM) and acts as a mediator of interaction between cells and the extracellular matrix and plays an important role in cell adhesion, migration, growth, and differentiation, thereby normalizing the body’s normal function (36). In the EMT process, which it happens tumor migration and proliferation., the expression of Vimentin and N-cad factors increases, and E- cadherin decreases (37). In a study to determine the expression of EMT markers in GC, Western blot was used and it was found that after overexpression of *FN1*, N-cad and Vimentin proteins are significantly less expressed, while E-cadherin showed a large increase, which indicates the relationship between *FN1* and EMT (38). Sun et al. (2020) reported about the relationship between *FN1* expression and clinical pathology and prognosis of GC that *FN1* is a potential biomarker for poor prognosis in patients with GC (39). Zang et al. (2017) reported that increased expression of miR-200c can prevent the proliferation, migration, and progression of GC by reducing the expression of the *FN1* gene (40). Low *FN1* expression leads to increased apoptosis and decreased cell proliferation by inactivating the PI3K/AKT signaling pathway (28).

Biglin (*BGN*) is an important member of the leucine-rich proteoglycan family that is involved in the development of various types of human cancer and their metastasis, and *BGN* is found in the extracellular matrix of various tissues (41). BGN can participate in the regeneration of blood vessels, the organization of the extracellular matrix, and the metabolic process of carbohydrates (41). *BGN* is an essential component of the extracellular matrix (ECM) and by encoding one of the ECM proteoglycans, it binds to TGF-beta, causing cancer (42). *BGN* has a significant negative correlation with B cells and a positive correlation with dendritic cells, CD8 + T and CD4 + T, macrophages, and neutrophils in various types of cancers including GC (43). Hu et al. (2014) reported that BGN regulates GC metastasis and plays an oncogenic role by activating the FAK signaling pathway (22). Chen et al. (2020) reported that BGN expression is significantly higher in GC tissues and is associated with lymph node metastasis, and depth of tumor invasion (44).

Thrombospondins (*THBS2*) play an important role in ECM receptor interaction pathways (27). *THBS2* are alglycoproteins released from various cells, including stromal fibroblasts, endothelial cells, and immune cells (45). The difference in *THBS2* expression in GC tissue was consistent with results that reported a vital role for the ECM receptor interaction pathway in cancer progression. Overexpression of *THBS2* is associated with cancer progression and metastasis in GC and can be used as a biomarker in predicting the clinical outcome of GC patients (46).

*SPARC* is a cysteine-rich acidic glycoprotein that acts as a mediator in tissue regeneration, expression of proteins involved in ECM, and formation of collagen and matrix metalloproteinases (13, 45). SPAR*C* expression in gastric cancer is significantly associated with metastasis and can be used as a useful marker in tumor prediction (47, 48).

The exact relationship between the hub gene and miRNA showed that the four genes *COL12A1, FN1, COL1A2*, and *COL5A2* target more miRNAs than the other hub genes, *SPARC, COL6A3, THBS2*, and *BGN.* Among these, the highest interaction was related to *COL12A1* (grade = 47) and the lowest interaction was related to BGN (grade =2). In cancer, miRNAs can generally act as tumor suppressors or oncogenes, and sometimes can even depending on the type of tumor play both roles (49). Also, miR-29a-3p, miR-101-3p, miR-183-5p, and miR-15a-5p were the top four interactive miRNAs, respectively, that targeted the most hub genes. miR-29a-3p reduces the proliferation and invasion of GC cells by regulating HAS3 expression (50, 51). On the other hand, MIAT uses the MIAT/miR-29a-3p/HDAC4 axis and by increasing MIAT expression causes high expression of HDAC4 as the downstream target of miR-29a-3p, thereby increasing cell proliferation, migration, and invasion of GC cells (52). By increasing EZH2 expression, LINC01303 can inhibit the activity of miR-101-3p, thereby inducing the proliferation, migration, and invasion of gastric cancer cells (53). One study showed that PHLDA1 expression is increased through the circ_0027599/miR-101 pathway, suppressing GC cells and their metastasis (54). miR-183-5p acts as an oncogene by reducing TPM1 expression and inactivating Bcl-2/P53 signaling pathways in GC, by increasing proliferation, migration, and cell invasion (55). Increased expression of miR-15a-5p reduces Gc cell metastasis. Studies have shown that LINC_00355 increases PHF19 expression, which targets miR-15a-5p downstream, resulting in the proliferation, transmission, and attack of cancer cells (56, 57).

Using the DGIdb database, a total of 6 drugs affecting GC were identified. The three drugs L19IL2, L19TNFA, and L19SIP 131I had the highest overall score on the website, with a large difference from the other drugs. All three of them belonged to the *FN1* gene. Excess B domain of fibronectin (ED-B) indicates tumor angiogenesis (58). L19, a human antibody, can target this marker and act as an immune cytokine when paired with IL2 (58). The results show that radiotherapy in combination with L19-IL2 will give a better response this response depends on the expression of ED-B (58, 59).

Intralesional administration of L19-IL2 and L19-TNF is a simple and effective way to eradicate non-surgical melanoma lesions or make them suitable for surgical resection. SIP, which is a small immunoprotein in combination with L19, can target ED-B (60). Tijink et al.(2006) Showed that radioimmunotherapy with L19-SIP-131I, alone or in combination with cetuximab, appeared to be effective in treating head and neck cancer (60).

## 5 Conclusions

As a result of this bioinformatics meta-analysis, eight hub genes including *COL1A2*, *FN1*, *BGN, THBS2, COL5A2, COL6A3, SPARC*, and *COL12A1* were identified that may play an important role in GC. They can be considered candidate genes in the GC prevention and early detection program. Further experimental studies are needed to confirm the findings of the present analysis. Also, miR-29a-3p, miR-101-3p, miR-183-5p, and miR-15a-5p were the top four interactive miRNAs, respectively, that targeted the most hub genes, and the three drugs of *L19IL2*, L19TNFA and L19SIP 131I had the highest overall score.

## Data availability statement

The original contributions presented in the study are included in the article/supplementary material. Further inquiries can be directed to the corresponding author.

## Ethics statement

Ethical review and approval was not required for the study on human participants in accordance with the local legislation and institutional requirements. Written informed consent for participation was not required for this study in accordance with the national legislation and the institutional requirements.

## Author contributions

ED and ZP: data analysis and writing the manuscript. SM and SG: designing, project administration, supervision, validation, review, and proofread. All authors have read and agreed to the published version of the manuscript. All authors contributed to the article.
